# Exome sequencing identifies a mutation in *TMC1* as a novel cause of autosomal recessive nonsyndromic hearing loss

**DOI:** 10.1186/s12967-016-0780-5

**Published:** 2016-01-28

**Authors:** Jiongjiong Hu, Fei Liu, Wenjun Xia, Lili Hao, Jun Lan, Zhenghua Zhu, Jing Ye, Duan Ma, Zhaoxin Ma

**Affiliations:** Department of Otorhinolaryngology, Shanghai East Hospital, Tongji University, Shanghai, 200032 People’s Republic of China; Key Laboratory of Metabolism and Molecular Medicine, Ministry of Education, Collaborative Innovation Center of Genetics and Development, Department of Biochemistry and Molecular Biology, Institute of Biomedical Sciences, School of Basic Medical Sciences, Fudan University, Shanghai, 200032 People’s Republic of China; Institutes of Biomedical Science, Fudan University, Shanghai, 200032 People’s Republic of China

**Keywords:** Deafness, Ear, Transmembrane channel-like 1, Hearing loss, Missense mutation

## Abstract

**Background:**

Autosomal recessive non-syndromic hearing loss (ARNSHL) is highly heterogeneous, and mutations in the gene encoding transmembrane channel-like 1 (TMC1) have been implicated in its development. To date, 35 homozygous mutations in *TMC1*, identified in over 60 families worldwide, have been shown to be associated with ARNSHL. However, few of these mutations were detected in the Chinese population. In this study, we describe a pathogenic missense mutation located in the T5–T6 domain of *TMC1* in a three-generation Chinese family with 14 members.

**Methods:**

Whole exome sequencing was performed using samples from one unaffected individual and two affected individuals to systematically search for deafness susceptibility genes. Candidate mutations and cosegregation of the phenotype were verified by polymerase chain reaction and Sanger sequencing in all of the family members.

**Results:**

We identified a novel *TMC1* mutation in exon 20, c.1979C>T, p.P660L, which segregated with prelingual autosomal recessive sensorineural hearing loss.

**Conclusions:**

We found a new missense mutation in the T5–T6 domain of *TMC1*, which is highly conserved in many species. These data support the potential conserved role of p.P660L in human *TMC1* function.

**Electronic supplementary material:**

The online version of this article (doi:10.1186/s12967-016-0780-5) contains supplementary material, which is available to authorized users.

## Background

Hearing loss (HL) is one of the most prevalent human birth defects, affecting one in 1000 individuals. Most congenital cases of HL have a genetic etiology, and nonsyndromic hearing loss (NSHL) accounts for approximately 80 % of genetic deafness [1]. To date, a total of 87 NSHL genes have been identified (http://hereditaryhearingloss.org), and most hearing impairments originating from these gene mutations are inherited in an autosomal recessive pattern. Genetic HL exhibits marked heterogeneity, which can be explained by the complexity of auditory system [2]. The proteins encoded by the identified deafness genes are involved in different functions, including gene regulation, embryonic development, ionic and osmotic homeostasis, synaptic transmission, generation of endocochlear potential, hair cell bundle morphology, and mechano-electrical transduction (MET) [3].

The gene encoding transmembrane channel-like 1 (*TMC1*) has been implicated in the pathogenesis of both dominant and recessive nonsyndromic HL, DFNA36 and DFNB7/11, respectively [4]. Although *TMC1* is expressed in cochlear hair cells and is involved in hair-cell function [5], the expression and function of the TMC1 protein in the inner ear are unclear. Deafness (dn) and Beethoven (Bth) mutant mice carrying recessive (Tmc1dn) and dominant (Tmc1Mhdabth) *TMC1* mutations show several physiological deficits in hair cell maturation, and recent studies have indicated that hair cells from Tmc1-knockout mice lack mechanosensory potential [5]. Chatzigeorgiou et al. [6] demonstrated that *TMC1* from *Caenorhabditis elegans* encodes a sodium-sensitive ion channel; however, this study lacked a mutant control, and the observations presented in this study have not been replicated for vertebrate *TMC1*. Another study found that *TMC1* is a component of hair cell transduction channels [7], although these results are controversial. Taken together, the results of many studies have supported that *TMC1* expression is required for conventional mechanotransduction in auditory and vestibular hair cells [7–9].

To date, 35 homozygous mutations in *TMC1*, identified in 60 families worldwide, have been shown to be associated with ARNSHL (Additional file 1: Table S1). However, these mutations have rarely been found in the Chinese population [2]. Almost all reported recessive cases have been shown to exhibit a similar phenotype characterized by prelingual severe-to-profound HL [3]. The most common recessive mutation in *TMC1* reported to affect HL is the nonsense mutation p.R34X, which accounts for over 30 % of mutant alleles of *TMC1* in Asian and North African populations [10].

In this study, we identified a novel homozygous mutation in *TMC1* located in exon 20, c.1979C>T, p.P660L in a three-generation, 14-member family (SH-02) presenting segregating ARNSHL with whole exome sequencing (WES). We further sequenced 500 ethnically unrelated healthy individuals and 300 sporadic deafness cases; none of these individuals carried the P660L mutation. Therefore, we concluded that the HL in this family was caused by novel homozygous mutation in *TMC1*.

## Methods

### Family recruitment and clinical evaluations

**Fig. 1:**
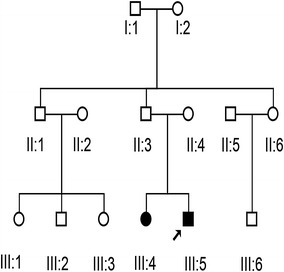
Pedigree of the Chinese family SH-02 with nonsyndromic autosomal recessive SNHL. *Open symbols* denote unaffected individuals; *filled black symbols* denote affected individuals. The *arrow* indicates the proband

A three-generation, non-consanguineous 14-member family (SH-02) presenting with segregating ARNSHL was identified by the Department of Otolaryngology, Head and Neck Surgery of Shanghai East Hospital, Tongji University, Shanghai, China (Fig. 1). All clinical information was collected at the Department of Otolaryngology and Head and Neck Surgery, Shanghai East Hospital, Tongji University, Shanghai, China. Medical histories were obtained using a questionnaire covering the following issues: subjective degree of HL, age at onset, progression, symmetry of hearing impairment, use of hearing aids, presence of tinnitus and vertigo, medication, noise and ototoxic drug exposure, pathological changes in the ear, and other relevant clinical manifestations. Systemic medical examinations and approximate intelligence assessments were also performed for all affected individuals. Another 300 individuals with sporadic HL participated in this study, in addition to 500 ethnically matched healthy controls. All procedures were approved by the Ethics Committee of Shanghai East Hospital, which is associated with Tongji University, and were carried out only after written informed consent had been obtained from all study participants and from the parents of participants younger than 18 years of age. Participants were informed that all data collected would be used only for scientific research and not for any commercial purposes.

### Audiological tests and imaging studies

Audiological tests were performed in a standard anechoic chamber with a pure-tone audiometer (Interacoustics AD229b; Interacoustics A/S DK-5610 Assens, Denmark) at frequencies ranging from 250 to 8000 Hz. Using an acoustic emmittance measurement apparatus (Interacoustics AT235h; Interacoustics A/S DK-5610 Assens), auditory brainstem response (ABR) was recorded ipsilaterally in response to click stimuli presented at 100 dBnHL (Interacoustics Eclipse EP2; Interacoustics A/S DK-5610 Assens). Additionally, a distortion product otoacoustic emissions system (DPOAE; 2f1–f2; Interacoustics DPOAE20 + TEb; Interacoustics A/S DK-5610 Assens Denmark) was used, where f2/f1 = 1.22; the level for f1 was 65 dB SPL, and the level for f2 was 50 dB SPL (DP S/N: 5 dB SPL). Ear endoscopy, computed tomography (CT) scans, and magnetic resonance imaging (MRI) were used to exclude deafness caused by anatomical abnormalities of the middle and inner ear.

### Whole exome sequencing

Genomic DNA was extracted from whole blood samples using a blood DNA kit (Qiagen, Germany), and 1 μg of purified gDNA was fragmented to 200 bp. End repair, adenylation, and adapter ligation were performed for library preparation according to the Illumina protocol. TruSeq DNA LT/HT Sample Prep Kit and TruSeq Exome Enrichment Kit were used to enrich exomes. Equal amounts of library samples were pooled and then hybridized to the customized capture array, including exons, splicing sites, and immediate flanking intron sequences. Sequencing was performed on an Illumina HiSeq 2500 to generate paired end reads. Reads that aligned to exon regions were collected for mutation identification and subsequent analysis. Samtools mpileup was used for variant calling and to identify SNPs and indels. ANNOVAR was used for annotating the genes.

### Sanger sequencing

Samples from all available members from the SH-02 family were subjected to Sanger sequencing to determine whether the potential mutations in causative genes, particularly the homozygous mutation in *TMC1*, cosegregated with the disease phenotype in this family. The polymerase chain reaction (PCR) products were sequenced using BigDye Terminator v3.1 Cycle Sequencing Kits (Applied Biosystems, Foster City, CA, USA) and analyzed using an ABI 3700XL Genetic Analyzer. And the primers for the TMC1 sequencing were as follows: TMC1-Forward: AGGATGGGCTCCTTCTTTGC; TMC1-Reverse: ACGAGGTTTCACCGTGTTGG.

### In silico analysis

In the present study, we used SIFT [11], Polyphen2 [12], and MutationTaster [13] software to determine possible changes in the protein structure that may affect the phenotype. Clustal X1.83 software was used to compare the human wild-type TMC1 protein sequence with orthologs from *Mus musculus*, *Physeter catodon*, *Macaca fiscicularis*, *Ailuropoda melanoleuca*, *Octodon degus*, and *Lipotes vexillifer* and to examine evolutionary conservation and structural prediction for this protein. Sequences were obtained from http://www.ensembl.org/.

## Results

### Clinical description

**Fig. 2:**
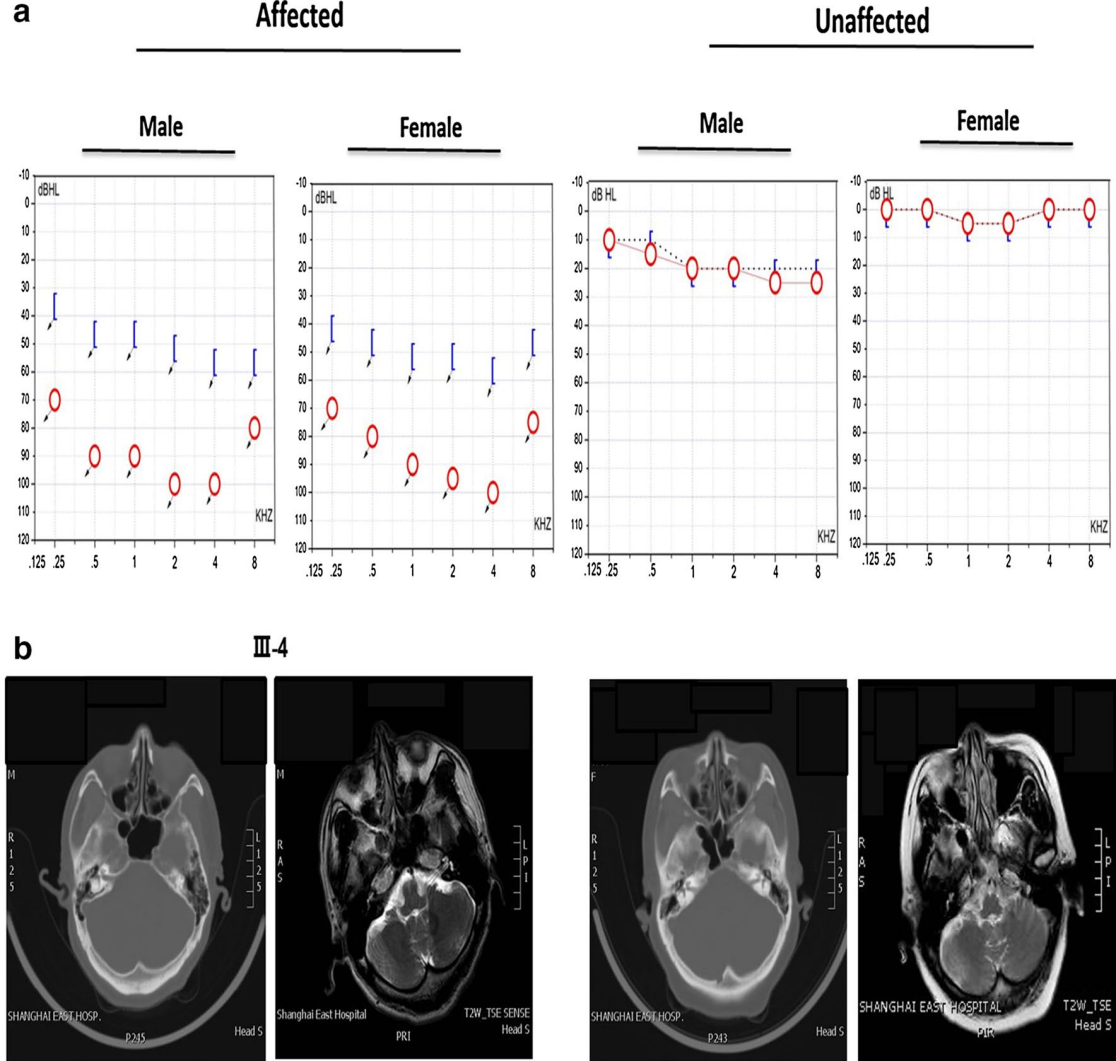
The SH-02 audiometric phenotype. **a** Pure-tone bone and air conduction thresholds are presented for the right ears of SH-02 family patients. **b** Based on the CT scan and MRI data of the proband (III5) and his sister (III4), the mastoid process and cochlea were well developed, and the ossicular chain was intact. Additionally, both the internal auditory meatus and the membranous labyrinth were well developed

The proband and his sister were congenital deaf-mute, while all their parents and grandparents had normal auditory and verbal functions. The two patients were delivered full-term after normal deliveries, and none of the parents had any history of constant exposure to noise or ototoxic drugs or a history of serious infection during pregnancy. Bone and air conduction of pure tone audiometry displayed no auditory reaction at any frequency. Tympanometry indicated a type A curve for the tympanogram, demonstrating that the tympanic cavity exhibited normal function, and acoustic reflex could not be induced in either ear (the threshold of the acoustic reflex was 100 dBHL; Fig. 2a). Otoacoustic emission could not be induced at any frequency. Upon ABR examination, well-differentiated wave profiles and regular latency could not be induced by click stimuli presented at 100 dBnHL in both ears, indicating that there was no effective acoustic information generated or sent to the brain by mechanosensory hair cells in the organ of Corti. CT scans and MRI data indicated that the mastoid process, cochlea, internal auditory meatus, and membranous labyrinth were all well developed, as was the ossicular chain (Fig. 2b, c). Clinical and instrumental evaluations did not reveal any evidence of syndromic features, such as cardiovascular diseases, diabetes, visual problems, or neurological disorders. The younger brother and his elder sister both displayed normal intelligence (Table 1).

**Table 1:** Summary of the audiological features of affected members of family SH-02

Patient	Gender	Age (years)		Use of aminoglycoside	Hearing test dBHL (PTA)		Verbal function	Level of hearing impairment	Vertigo	Tinnitus	Intelligence
		At testing	At onset		Left ear	Right ear					
III-4	Female	35	On birth	No	Null	Null	Null	Anacusia	No	No	Normal
III-5	Male	31	On birth	No	Null	Null	Null	Anacusia	No	No	Normal

### Whole exome sequencing

To systematically search for deafness susceptibility genes, we performed WES of samples from one unaffected (II-3) and two affected (III-4 and III-5) individuals from the SH-02 family pedigree. An average of 4.67 billion bases of high-quality sequence was generated per individual, with an average sequencing depth of approximately 97 in the target region; this satisfied the requirements for calling single nucleotide polymorphisms (SNPs) and indels. The sequencing data were aligned to the NCBI human reference genome and compared with dbSNP138, which contains pilot data from the 1000 Genomes Project, from eight sequenced HapMap individuals and from ten individuals in the YH database.

In total, 7898 variants were identified in the two patients; 5760 of these were nonsynonymous variants, including splice acceptor and donor site mutations and coding indels, which were more likely to be pathogenic mutations. Next, these variants were prioritized for further evaluation using two filtering criteria: (1) variants within the allele frequency cutoff (less than 0.01 in the dbSNP138, HapMap, 1000 Genomes, and local datasets); and (2) variant found in all the affected individuals but not in the unaffected individual; variants meeting these criteria were retained for further analysis. These filtering criteria reduced the list of candidate variants to 25 nonsynonymous homozygous variations.

**Fig. 3:**
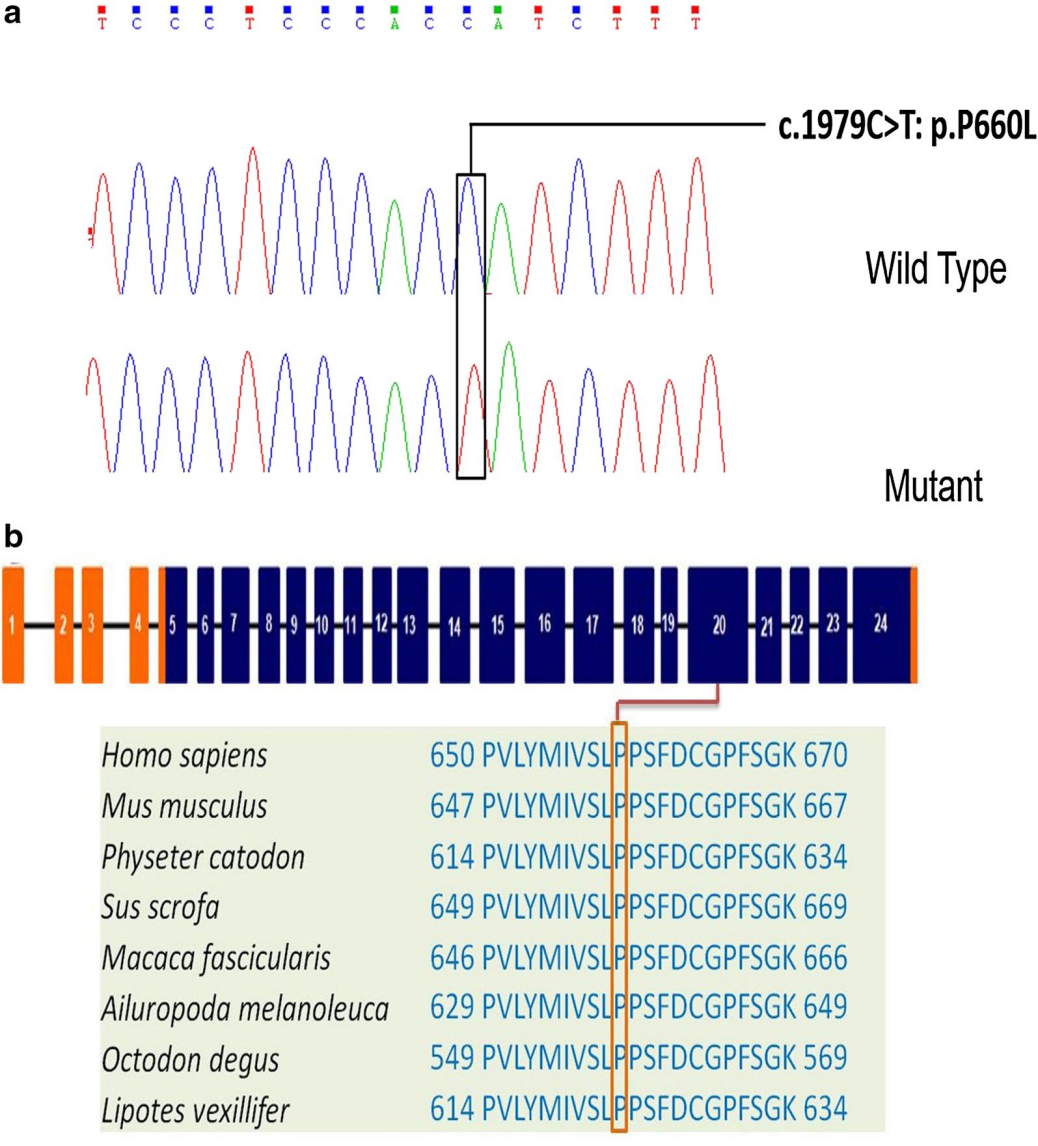
Mutation analysis of the Chinese family SH-02. **a** DNA sequences of homozygous missense c.1979C>T mutations and the wild-type control. **b** The structure of TMC1A depicts the position of c.1979C>T, a mutation in exon 20 and p.P660L in TM2 domain

Then, we screened 25 variations found among the pedigree samples by Sanger sequencing and found a missense variant, c.1979C>T, p.P660L, in exon 20 of TMC1 [NM_138691, (MIM^#^600974)], which co-segregated with the disease (Fig. 3a). This novel *TMC1* mutation was exclusively identified in all two affected patients but was not found in the 12 unaffected family members. To assess the possibility that P660L is a disease-causing mutation, we further sequenced 500 ethnically unrelated healthy individuals and 300 sporadic deafness cases, and confirmed that none of these people carried the P660L mutation. Thus, our data suggested that P660L was a disease-causing mutation in the Chinese pedigree (SH-02) with NSHL.

### In silico analysis

To determine the potential effects of the p.P660L missense mutation on TMC1 function, we further performed in silico analyses. This mutation was predicted to be “Damaging”, “Probably Damaging”, and “Disease-causing” by SIFT, Polyphen2, and MutationTaster, respectively (Table 2). The conservation analysis indicated that the Pro residue at 660 in the TMC1 protein was conserved across *Homo sapiens*, *Mus musculus*, *Physeter catodon*, *Macaca fiscicularis*, *Ailuropoda melanoleuca*, *Octodon degus*, and *Lipotes vexillifer* (Fig. 3b). This finding indicated that this novel mutation may be the cause of the observed HL in this Chinese family.

**Table 2:** A novel variant in the proband of family SH-02

Gene	MIM no.	Nucleotide	Amino acid	Zygosity	Prediction information		
*TMC1*	138691	c.1979C>T	p.P660L	Hom	SIFT	Polyphen2	MutationTaster
					Damaging	Probably damaging	Disease causing

## Discussion

The World Health Organization has estimated that 360 million people worldwide have disabling HL (http://www.who.int/mediacentre/factsheets/fs300/en/) and that as the population ages, the global burden of diseases attributable to deafness will increase [14]. Linkage analysis was once regarded as the most powerful and widely used method for linking critical intervals to identify disease-causing genes in large pedigrees; however, this method is not appropriate for small families. Recently, with the marked advancements in sequencing technology, WES, which involves targeted sequencing of the protein-coding subset of the human genome, has been used as a convenient, rapid method for identification of new genes. Next-generation sequencing has advantages of small samples quantity, minimal cost, high-throughput sequencing, and low requirement for family size [15]. In this study, WES was used to find the disease-causing gene of a Chinese family with HL, and we identified a novel *TMC1* missense mutation in exon 20, c.1979C>T, p.P660L.

**Fig. 4:**
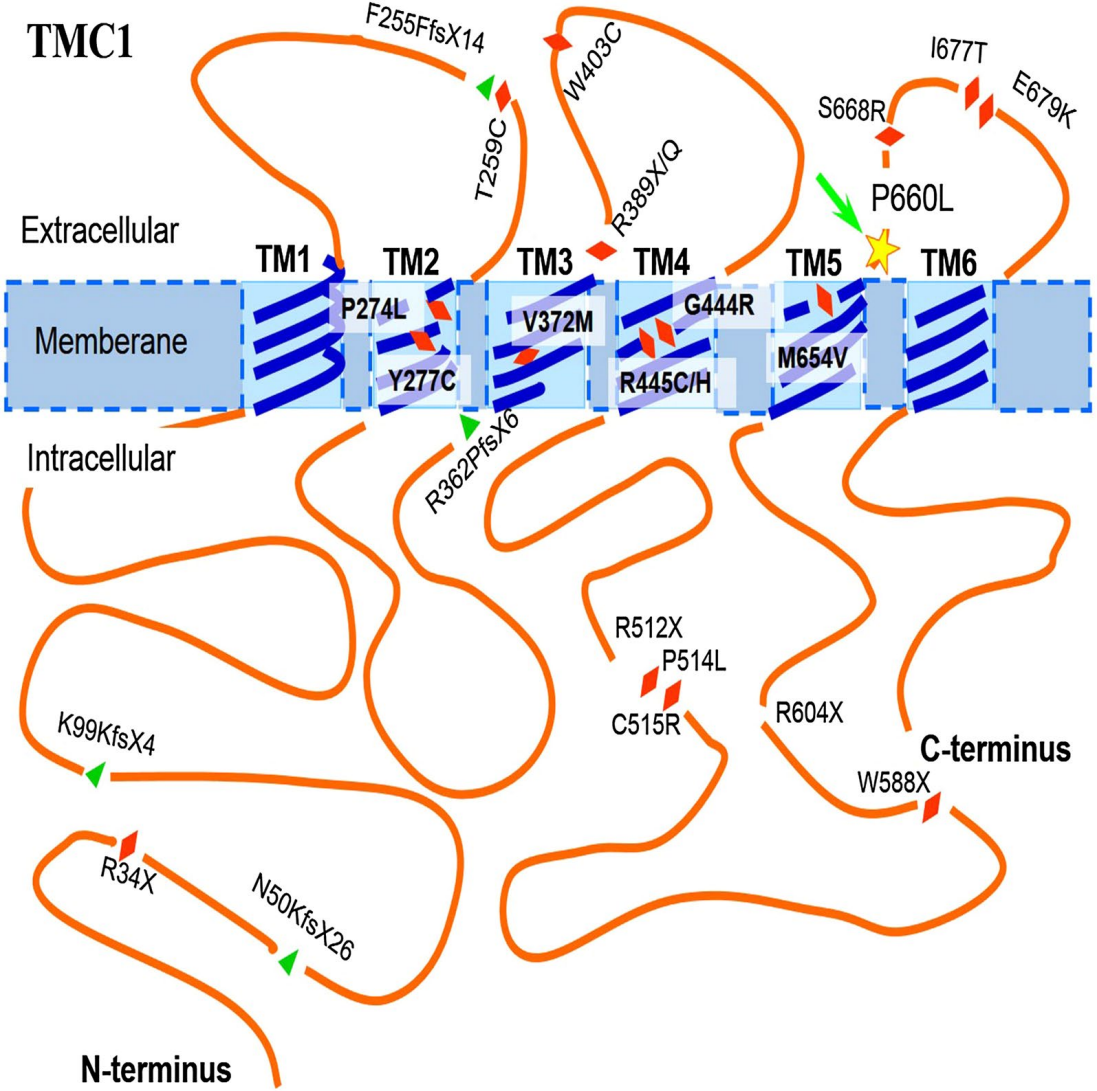
Location of the p.P660L mutation and other reported exonic mutations in *TMC1*. The *red rhombus* represents a missense or nonsense mutation; the *green triangle* represents a deletion mutation; and the *green arrow*/*yellow star* represents the p.P660L mutation located within a predicted third extracellular loop found between the fifth and sixth transmembrane domains

*TMC1* on chromosome 9q21 contains 24 exons that make up a coding region of 2283 nucleotides. The gene sequence is highly conserved, which suggests strong selective pressure throughout animal evolution. Mutations in *TMC1* are a common cause of autosomal recessive nonsyndromic deafness, particularly in India, Pakistani, Turkish, and Tunisian families [2]. Thirty-five reported homozygous recessive mutations in *TMC1*, found in over 60 families worldwide, have been identified in different parts of *TMC1* and cause different structural and functional disparity in intracellular and extracellular domains (Fig. 4).

*TMC1* topology is predicted to include six membrane-spanning domains with three extracellular loops, a large intracellular loop settled between transmembrane (TM) domains four and five, a long intracellular N-terminus, and a short intracellular C-terminus. The structure suggests that the protein may function as a receptor, transporter, pump, or channel [16]. The mutation found in this study, p.P660L, was located within a predicted third extracellular loop situated between the fifth and sixth TM domains (Fig. 4). Pro660 is located in a highly conserved glycosyltransferase domain, which is highly conserved among many species. To the best of our knowledge, this is the first report describing a missense mutation in the glycosyltransferase domain of *TMC1*, supporting the importance of the conserved role of P660L in human TMC1 function.

*TMC1* expression is constant in mature cochlear and vestibular hair cells, as shown in dn and Bth mutant mice carrying recessive (Tmc1dn) and dominant (Tmc1bth) *TMC1* alleles. In the DFNA36 models, heterozygous mice (*Tmc1*^*Bth/*+^) showed progressive hair cell degeneration [17], while homozygous mice (*Tmc1*^*Bth/Bth*^) exhibited profound deafness [5]. Models of human recessive deafness, i.e., DFNB7/11, *TMC1*^*dn/dn*^ mice, do not have cochlear responses to sound stimuli and show several physiological deficits in hair cell maturation [5]. Kim et al. [9] concluded that *TMC1*/2 double mutant mice lacked conventional mechanotransduction, leading them to hypothesize that TMC1 is required for targeting the MET channel to the tips of the stereocilia, where they can interact with other constituents of the transduction complex, including the tip link. Although the specific function of TMC1 is unknown, recent studies have revealed that TMC1 is necessary for MET in cochlear and vestibular hair cells. Additionally, TMC1 is thought to be a component of the mechanotransduction channel in hair cells of the mammalian inner ear [3].

*Caenorhabditis eleganstmc1* has been shown to encode a sodium-sensitive ion channel. The predicted structure of TMC1 is similar to that of the α-subunit of voltage-dependent K^+^ channels, which have six TM segments and intracellular N- and C-termini [18], and TMC1 has been predicted to function as an ion channel or transporter mediating K^+^ homeostasis in the inner ear [19]. Ion channels serve many functions, including the transport of ions and water, control of electrical excitability, and regulation of ion homeostasis. The first four TM domains of the K^+^ channel α-subunit act as voltage sensors for activation gating [20], whereas the intervening segment between TM5 and TM6 appears to confer channel function [18]. TMC1 c.1979C>T, p.P660L is located in a predicted third extracellular loop located extracellularly between TM5 and TM6, and mutations in this loop may affect ion channel function in hair cells.

## Conclusion

In this report, we described a novel missense mutation in the highly conserved T5–T6 domain of *TMC1*, suggesting the potentially conserved role of Pro660 in the human amino acid sequence. This is only the second homozygous mutation in *TMC1* reported in the Chinese population. Moreover, this novel mutation expands the mutational spectrum of *TMC1*, which will contribute to the clinical understanding of HL caused by mutations in this gene.

## Additional file

10.1186/s12967-016-0780-5 Summary of reported homozygous TMC1 mutations.
