# Supplementary material for: Exome sequencing identifies a mutation in TMC1 as a novel cause of autosomal recessive nonsyndromic hearing loss
Source: J Transl Med. 2016 Jan 28;14:29. doi: 10.1186/s12967-016-0780-5 (PMC4731951; doi:10.1186/s12967-016-0780-5)
Supplement: Supplementary file 1 — 10.1186/s12967-016-0780-5 Summary of reported homozygous TMC1 mutations. [file 12967_2016_780_MOESM1_ESM.docx]

**S1 Table Clinical phenotypes of hearing loss patients caused by *TMC1* various mutations.**

| NO. | Family | Origin | Nucleotide mutation^a^ | Predicted effect | Exon/Intron number | Onset of HL | Severity of HL | Domain | Reference |
| --- | --- | --- | --- | --- | --- | --- | --- | --- | --- |
| 1 | PKDF22 | Pakistan | c.-195_16deld | Genomic deletion | exons 4–5 and adjacent introns | Prelingual | Severe to profound |  | ([1](#_ENREF_1)) |
| 2 | PKDF274 | Pakistan | c.16+1G>T | Splice disruption | Intron 5 | Prelingual | Severe to profound |  | ([2](#_ENREF_2)) |
| 3 | 734, 763 | Turkey | c.64+2T>A | Splice disruption | Intron 6 | Congenital/Prelingual | Profound |  | ([3](#_ENREF_3)) |
| 4 | PKSR9, PKSN9, PKSN24, PKDF7,PKDF75 | Pakistan | c.100C>T | p.R34X | Exon 7 | Prelingual | Severe to profound | N-teminus | ([1](#_ENREF_1)) |
|  | PKDF69, PKDF178,PKDF243, PKDF319,PKDF401 | Pakistan | c.100C>T | p.R34X | Exon 7 | Prelingual | Severe to profound |  | ([2](#_ENREF_2)) |
|  | Family A, B, C | Tunisia | c.100C>T | p.R34X | Exon 7 | Congenital | Profound |  | ([4](#_ENREF_4)) |
|  | 935 | Iran | c.100C>T | p.R34X | Exon 7 | Congenital | Severe to profound |  | ([5](#_ENREF_5)) |
|  | Nas | Lebanon/Jordan | c.100C>T | p.R34X | Exon 7 | Congenital | Severe to profound |  | ([5](#_ENREF_5)) |
|  | 685 | Turkey | c.100C>T | p.R34X | Exon 7 | Congenital/Prelingual | Severe to profound |  | ([6](#_ENREF_6)) |
| 5 | L787 | Iran | c.150delT | p.N50KfsX26 | Exon 7 | Congenital | Profound | N-teminus | ([7](#_ENREF_7)) |
| 6 | M36 | Iran | c.236+1G>A | Splice disruption | Intron 7 | Congenital | Severe to profound |  | ([5](#_ENREF_5)) |
| 7 |  | India | 237-6T>G | Splice disruption | Intron 7 | Prelingual | Severe to profound |  | ([8](#_ENREF_8)) |
| 8 | IN-DKB6 | North America (Indian) | c.295delA | p.K99KfsX4 | Exon 8 | Prelingual | Severe to profound | N-teminus | ([1](#_ENREF_1)) |
| 9 | 11DF | Pakistan | c.362+18A>G | p.Glu122Tyrfs*10 | Intron 8 | Congenital | Severe to profound |  | ([9](#_ENREF_9)) |
| 10 | PKSR25 | Pakistan | c.536-8T>A | Splice disruption | Intron 10 | Prelingual | Severe to profound |  | ([1](#_ENREF_1)) |
|  | 4090 | Pakistan | c.536-8T>A | Splice disruption | Intron 10 | Prelingual | Severe to profound |  | ([10](#_ENREF_10)) |
| 11 | DF139 | Turkey | c.767delT | p.F255FfsX14 | Exon 13 | Congenital | Severe to profound | T1-T2 | ([5](#_ENREF_5)) |
| 12 | TR56 | Turkey | c.776A>G | p.T259C | Exon 13 | Prelingual | Profound | T1-T2 | ([11](#_ENREF_11)) |
| 13 | TR47 | Turkey | c.821C>T | p.P274L | Exon 13 | Prelingual | Profound | T2 | ([11](#_ENREF_11)) |
| 14 | 4049 | Pakistan | c.830A>G | p.Y277C | Exon 13 | Prelingual | Severe to profound | T2 | ([10](#_ENREF_10)) |
| 15 | PKSR1a | Pakistan | c.884+1G>A | Splice disruption | Exon 13 | Prelingual | Severe to profound |  | ([1](#_ENREF_1)) |
| 16 | TR50 | Turkey | c.1083_1087delCAGAT | p.R362PfsX6 | Exon 15 | Prelingual | Profound | T2-T3(deletion of 5 domains) | ([11](#_ENREF_11)) |
| 17 | 4119, 4160 | Pakistan | c.1114G>A | p.V372M | Exon 15 | Prelingual | Severe to profound | T3 | ([10](#_ENREF_10)) |
| 18 | Family D | Tunisia | c.1165C>T | p.R389X | Exon 15 | Congenital | Profound | T3-T4 | ([4](#_ENREF_4)) |
|  | Fay | Lebanon/Jordan | c.1165C>T | p.R389X | Exon 15 | Congenital | Severe to profound |  | ([5](#_ENREF_5)) |
| 19 | DF135 | Turkey | c.1166G>A | p.R389Q | Exon 15 | Congenital | Severe to profound | T3-T4 | ([5](#_ENREF_5)) |
| 20 | D555 | China | c.1209G>C | p.W403C | Exon 15 | Prelingual | Severe to profound | T3-T4 | ([12](#_ENREF_12))] |
| 21 | 232 | Turkey | c.1330G>A | p.G444R | Exon 16 | Congenital/Prelingual | Profound | T4 | ([6](#_ENREF_6)) |
| 22 | 647 | Turkey | c.1333C>T | p.R445C | Exon 16 | Congenital/Prelingual | Severe to profound | T4 | ([6](#_ENREF_6)) |
| 23 | TR63 | Turkey | c.1334G>A | p.R445H | Exon 16 | Prelingual | Profound | T4 | ([11](#_ENREF_11)) |
| 24 | PKSR20a | Pakistan | c.1534C>T | p.R512X | Exon 17 | Prelingual | Severe to profound | T4-T5 | ([1](#_ENREF_1)) |
| 25 | PKDF431 | Pakistan | c.1541C>T | p.P514L | Exon 17 | Prelingual | Severe to profound | T4-T5 | ([2](#_ENREF_2)) |
| 26 | PKDF329,PKDF511 | Pakistan | c.1543T>C | p.C515R | Exon 17 | Prelingual | Severe to profound | T4-T5 | ([2](#_ENREF_2)) |
| 27 | W06-792 | Netherlands | c.1763+3A>G | p.W588WfsX81 | Intron 19 | Post-lingual | Profound 1st decade:highfrequencies, 2nd-3rd decade: all frequencies |  | ([13](#_ENREF_13)) |
| 28 | Family E | Tunisia | c.1764G>A | p.W588X | Exon 20 | Congenital | Profound | T4-T5 | ([4](#_ENREF_4)) |
| 29 | GRE | Greece | c.1810C>T | p.R604X | Exon 20 | Congenital | Severe to profound | T4-T5 | ([5](#_ENREF_5)) |
| 30 | IN-M17 | North America (Indian) | c.1960A>G | p.M654V | Exon 20 | Prelingual | Severe to profound | T5 | ([1](#_ENREF_1)) |
| 31 | SH-02 | China | c.1979C>T | p.P660L | Exon 20 | Congenital | Profound | T5-T6 | This study |
| 32 | 4070, 4138 | Pakistan | c.2004T>G | p.S668R | Exon 21 | Prelingual | Severe to profound | T5-T6 | ([10](#_ENREF_10)) |
|  | PKDF419 | Pakistan | c.2004T>G | p.S668R | Exon 21 | Prelingual | Severe to profound |  | ([2](#_ENREF_2)) |
| 33 | 675 | Turkey | c.2030T>C | p.I677T | Exon 21 | Congenital/Prelingual | Profound | T5-T6 | ([6](#_ENREF_6)) |
| 34 | 4008, 4033 | Pakistan | c.2035G>A | p.E679K | Exon 21 | Prelingual | Severe to profound | T5-T6 | ([10](#_ENREF_10)) |
| 35 | DF56 | Tunisia | 2260+2T>A | Splice disruption | Intron23 |  | Severe to profound |  | ([14](#_ENREF_14)) |
| 36 | 551 | Turkey | c.1696_2283del^e^ | Genomic deletion |  | Congenital/Prelingual | Profound |  | ([6](#_ENREF_6)) |

**S1 Table. Summary of reported homozygous TMC1 mutations.**

This table summarizes all reported homozygous mutations in TMC1 with population information and clinical details (congenital/prelingual/postlingual onset, severity, progressive or stable hearing loss) along with domain positions of the exonic mutations. After systematic analysis of the mutations reported in previous studies, we found no significant genotype/phenotype correlations.

1. Kurima K, Peters LM, Yang Y, Riazuddin S, Ahmed ZM, Naz S, et al. Dominant and recessive deafness caused by mutations of a novel gene, TMC1, required for cochlear hair-cell function. Nature genetics. 2002;30(3):277-84.

2. Kitajiri SI, McNamara R, Makishima T, Husnain T, Zafar AU, Kittles RA, et al. Identities, frequencies and origins of TMC1 mutations causing DFNB7/B11 deafness in Pakistan. Clinical genetics. 2007;72(6):546-50.

3. Nakanishi H, Kurima K, Kawashima Y, Griffith AJ. Mutations of TMC1 cause deafness by disrupting mechanoelectrical transduction. Auris Nasus Larynx. 2014;41(5):399-408.

4. Tlili A, Rebeh IB, Aifa-Hmani M, Dhouib H, Moalla J, Tlili-Chouchene J, et al. TMC1 but not TMC2 is responsible for autosomal recessive nonsyndromic hearing impairment in Tunisian families. Audiology & neuro-otology. 2008;13(4):213-8.

5. Hilgert N, Alasti F, Dieltjens N, Pawlik B, Wollnik B, Uyguner O, et al. Mutation analysis of TMC1 identifies four new mutations and suggests an additional deafness gene at loci DFNA36 and DFNB7/11. Clinical genetics. 2008;74(3):223-32.

6. Sirmaci A, Duman D, Ozturkmen-Akay H, Erbek S, Incesulu A, Ozturk-Hismi B, et al. Mutations in TMC1 contribute significantly to nonsyndromic autosomal recessive sensorineural hearing loss: a report of five novel mutations. International journal of pediatric otorhinolaryngology. 2009;73(5):699-705.

7. Yang T, Kahrizi K, Bazazzadeghan N, Meyer N, Najmabadi H, Smith RJ. A novel mutation adjacent to the Bth mouse mutation in the TMC1 gene makes this mouse an excellent model of human deafness at the DFNA36 locus. Clinical genetics. 2010;77(4):395-8.

8. Ganapathy A, Pandey N, Srisailapathy CRS, Jalvi R, Malhotra V, Venkatappa M, et al. Non-Syndromic Hearing Impairment in India: High Allelic Heterogeneity among Mutations in TMPRSS3, TMC1, USHIC, CDH23 and TMIE. PloS one. 2014;9(1).

9. Shafique S, Siddiqi S, Schraders M, Oostrik J, Ayub H, Bilal A, et al. Genetic spectrum of autosomal recessive non-syndromic hearing loss in Pakistani families. PloS one. 2014;9(6):e100146.

10. Santos RL, Wajid M, Khan MN, McArthur N, Pham TL, Bhatti A, et al. Novel sequence variants in the TMC1 gene in Pakistani families with autosomal recessive hearing impairment. Human mutation. 2005;26(4):396.

11. Kalay E, Karaguzel A, Caylan R, Heister A, Cremers FP, Cremers CW, et al. Four novel TMC1 (DFNB7/DFNB11) mutations in Turkish patients with congenital autosomal recessive nonsyndromic hearing loss. Human mutation. 2005;26(6):591.

12. Yang T, Wei X, Chai Y, Li L, Wu H. Genetic etiology study of the non-syndromic deafness in Chinese Hans by targeted next-generation sequencing. Orphanet journal of rare diseases. 2013;8:85.

13. de Heer AM, Collin RW, Huygen PL, Schraders M, Oostrik J, Rouwette M, et al. Progressive sensorineural hearing loss and normal vestibular function in a Dutch DFNB7/11 family with a novel mutation in TMC1. Audiology & neuro-otology. 2011;16(2):93-105.

14. Riahi Z, Bonnet C, Zainine R, Louha M, Bouyacoub Y, Laroussi N, et al. Whole Exome Sequencing Identifies New Causative Mutations in Tunisian Families with Non-Syndromic Deafness. PloS one. 2014;9(6).
